# Implementation and assessment of a postreprocessing endoscope surveillance program at the University of Kentucky, 2019–2024

**DOI:** 10.1017/ice.2026.10449

**Published:** 2026-06

**Authors:** Court Desmond, Faith Fursman, Derek Forster, Julie A. Ribes, Sandra L. Mills, Kimberly Blanton, Kevin Hatton, Rachel Howard, David Olafsson, Sean McTigue, Nicholas Van Sickels, Sherese Hinton, Deborah R. Flomenhoft, Takaaki Kobayashi

**Affiliations:** 1 College of Medicine, University of Kentucky, USA; 2 Department of Pharmacy Practice and Service, College of Pharmacy, University of Kentucky, Lexington, KY, USA; 3 Infection Prevention and Control, University of Kentucky, Lexington, USA; 4 Division of Infectious Diseases, University of Kentucky, Lexington, USA; 5 Department of Pathology and Laboratory Medicine, University of Kentucky, Lexington, USA; 6 Department of Anesthesiology, University of Kentucky, Lexington, USA; 7 Division of Digestive Disease and Nutrition, University of Kentucky, Lexington, USA

## Abstract

We evaluated an endoscope surveillance culture program at a tertiary academic center from 2019–2024. Postreprocessing culture positivity was highest for esophagogastroduodenoscopy (25.9%). Carbapenem-resistant organism matches between endoscope and patient isolates occurred in 5% of positive cultures.

## Background

The risk of bacterial transmission through gastrointestinal endoscopes remains a critical concern in healthcare-associated infections, driven by the complex design of endoscopes and potential lapses in reprocessing protocols.^1^ Contaminated endoscopes can transmit multidrug-resistant organisms, endangering patient safety.^2^ Microbial sampling and culturing of endoscopes postreprocessing for multidrug-resistant organisms, such as carbapenem resistant organisms (CRO), can evaluate the effectiveness of reprocessing protocols and minimize patient risk of hospital-acquired infection. Current U.S. guidelines for endoscope reprocessing do not mandate routine surveillance sampling; however, some facilities have adopted routine or periodic cultures to monitor reprocessing efficacy. The Centers for Disease Control and Prevention (CDC) has published protocols to support such practices, emphasizing the need to identify persistent transmission risks.^3^

## Methods

We conducted a retrospective study at the University of Kentucky Healthcare (UKHC) from January 1, 2019 to June 30, 2024. UKHC implemented an active surveillance program in July 2016 to monitor reprocessing efficacy across four types of gastrointestinal endoscopes: endoscopic retrograde cholangiopancreatography (ERCP) scopes, esophagogastroduodenoscopy (EGD) scopes, endoscopic ultrasound (EUS) scopes, and colonoscopy scopes. ERCP scopes were subjected to weekly surveillance cultures, while targeted cultures were performed on all scope types following procedures on patients colonized with carbapenem resistant organisms (CRO). A total of 107 endoscopes were included in this study: 16 ERCP scopes, 41 EGD scopes, 19 EUS scopes, and 31 colonoscopes. To be included in the analysis, each culture had to be performed between January 1, 2019, and June 30, 2024. For cultures associated with carbapenem-resistant organism (CRO) investigations, patient-identifiable information (eg, legal name or MRN) and documented CRO data were required.

Postreprocessing sampling was conducted in accordance with manufacturer instructions for use (IFU) and institutional protocols. Randomly selected scopes were cultured after reprocessing. Scopes with positive cultures underwent subsequent reprocessing and culturing. This was all performed prior to patient reuse. Organisms were classified as “concerning” or “non-concerning” based on published CDC criteria.^4,5^ A “match” was defined as isolation of the same organism from both a patient and a scope, with both isolates classified as carbapenem-resistant (Supplemental material,^6^).

## Results

**Figure 1.**
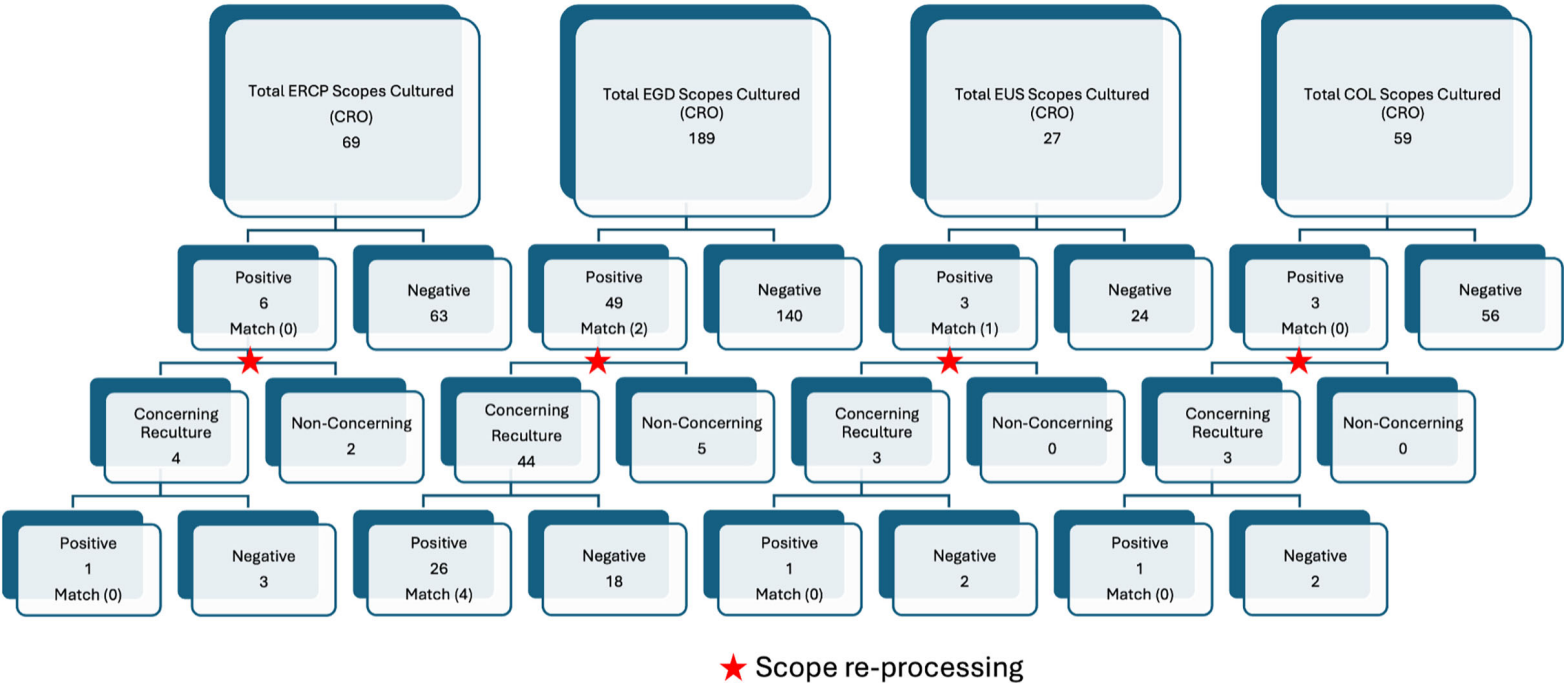
Figure 1 long description. CRO surveillance for ERCP cultured scopes, EGD cultured scopes, EUS cultured scopes, COL cultured scopes.

**Figure 2.**
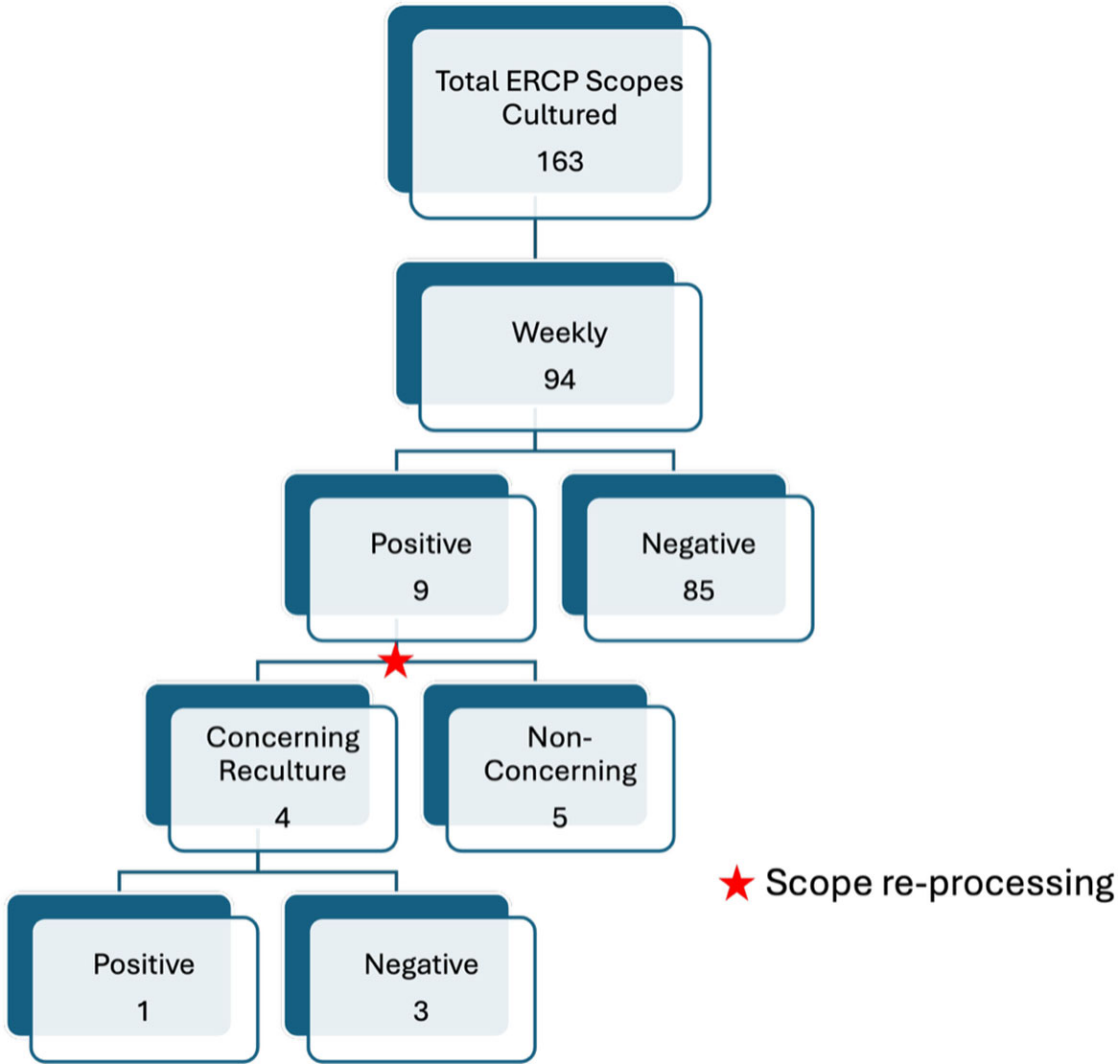
Figure 2 long description. Weekly surveillance of ERCP cultured scopes.

ERCP scopes were cultured 163 times during the study period, including 94 cultures through weekly surveillance and 69 cultures through CRO-targeted surveillance (Figures 1 and 2). Weekly cultures revealed a 9.6% positivity rate (9/94), while targeted cultures showed an 8.7% positivity rate (6/69). No matching CRO was found between contaminated ERCP scopes and patient isolates in either group.

EGD scopes were cultured 189 times for targeted CRO surveillance, 49 tested positive, corresponding to a positivity rate of 25.9%. Of these 49 positive EGD cultures, 2 (4.1%) yielded organisms matching patient CRO isolates.

EUS scopes were cultured 27 times for targeted CRO surveillance, with a positivity rate of 11.1% (3/27). Notably, 1 of the 3 contaminated EUS scopes (33.3%) had a matching isolate to a patient colonized with a CRO.

Colonoscopy scopes were cultured 59 times for targeted CRO surveillance, 3 tested positive (5.1%), with no matches to patient isolates identified.

## Discussion

This study evaluated the utility of an institutional endoscope surveillance culture program by examining postreprocessing contamination and CRO match rates across four commonly used gastrointestinal scope types. EGD demonstrated the highest overall positivity rate at 25.9%, whereas EUS exhibited the highest rate of matching to patient isolates at 33.3%. Although ERCP scopes are widely recognized as high-risk for contamination due to their complex duodenoscope design, our findings underscore that other scope types, such as EGD and EUS, may also harbor clinically significant contamination despite adherence to IFU-based reprocessing protocols. Overall, matched CRO isolates were identified in 5% (3 of 61) of positive cultures obtained following initial reprocessing.

The literature on endoscope-associated infections (EAIs) has predominantly focused on ERCP procedures, in part due to well-documented challenges with duodenoscope reprocessing.^7^ A meta-analysis reported a 15.25% contamination rate among reprocessed duodenoscopes.^8^ In our study, ERCP scope positivity rates were lower than the rates for EGD scopes. These findings suggest that limiting surveillance efforts to ERCP alone may overlook contamination risks associated with other gastrointestinal endoscopes. Differences in surveillance frequency across scope types may have influenced observed positivity rates. ERCP scopes underwent routine weekly surveillance, whereas other scope types were primarily cultured during CRO investigations. Additionally, increased institutional awareness surrounding duodenoscope-associated infections may have resulted in more rigorous reprocessing oversight for ERCP scopes compared to other devices.^9^

Several factors likely contribute to the observed variation in contamination rates. These include scope design complexity, differences in manual cleaning techniques among technicians, variability in automated endoscope reprocessor performance, and environmental conditions affecting scope drying, storage, and transport.^7^ While our institution maintained adherence to IFU and internal protocols, unmeasured deviations in practice or equipment may have contributed to residual contamination. Additionally, the possibility of contamination during postreprocessing handling or storage—both of which occur in non-sterile environments—cannot be excluded.

Although the match rate between contaminated scopes and patient isolates was low across most scope types, the presence of CROs on reprocessed endoscopes remains clinically meaningful. The detection of such organisms, even in the absence of direct transmission, indicates a breach in disinfection integrity and a potential risk to future patients. A previously published review estimated that composite EAI rates following gastrointestinal procedures were approximately 0.2%, with higher rates following ERCP (0.8%), followed by upper GI endoscopy (0.123%) and lower GI endoscopy (0.073%).^10^ While these rates are relatively low, they translate into substantial absolute numbers given the volume of procedures performed nationwide.

At our institution, any scope found to be contaminated with concerning organisms underwent immediate reprocessing and was quarantined until clearance. If repeat cultures remained positive, the scope was returned to the manufacturer for further inspection, in accordance with Food and Drug Administration (FDA) recommendations. The FDA has increasingly advocated for the adoption of disposable or partially disposable endoscope components in response to persistent contamination and EAIs.^11^ However, the routine use of disposable devices remains limited due to their high cost, environmental burden, and lack of availability across all scope types. Consequently, routine surveillance cultures may serve as an effective intermediary solution to identify contamination and prevent patient harm.

This study has several limitations. First, we did not assess patient transmission events, as contaminated scopes were not reused before undergoing repeat reprocessing. Second, the manual cleaning phase was not systematically audited, which could contribute to unrecognized variability in scope decontamination. Third, postreprocessing contamination during transport or storage could not be definitively excluded. Lastly, the study was conducted at a single academic center with specific workflows and equipment, which may limit generalizability to other institutions.

While postreprocessing contamination was observed across multiple scope types, CRO matches were uncommon. However, we did not systematically evaluate patient transmission events, limiting conclusions regarding the clinical impact of contamination. Given the low match rate observed in this single-center study, universal surveillance culturing may not be warranted across all settings. A risk-based or targeted surveillance strategy may be more appropriate, particularly for higher-risk scope types or in institutions with endemic multidrug-resistant organisms.

## Supporting information

10.1017/ice.2026.10449.sm001 Desmond et al. supplementary material Desmond et al. supplementary material

## Supplementary material

The supplementary material for this article can be found at https://doi.org/10.1017/ice.2026.10449.

## Figure 1 Long description

The bar graph compares the number of scopes cultured for different types of endoscopes, including ERCP, EGD, EUS, and COL, and their test results. The graph features four main categories, each with a total number of scopes cultured: ERCP with 69 scopes, EGD with 189 scopes, EUS with 27 scopes, and COL with 59 scopes. Each category is further divided into positive and negative results, with subcategories for concerning and non-concerning recultures. The graph highlights the number of positive and negative results, as well as the number of concerning and non-concerning recultures for each type of endoscope. It also indicates instances of scope reprocessing with red stars. The x-axis represents the types of endoscopes and their subcategories, while the y-axis represents the number of scopes. The color scheme includes shades of blue and gray, with red stars indicating scope reprocessing. All values are approximated.

Navigate back to Figure 1.

## Figure 2 Long description

The bar graph compares the number of ERCP scopes cultured weekly, with a total of 163 scopes. It shows that 94 scopes were cultured weekly, with 9 testing positive and 85 testing negative. Of the positive cases, 4 were concerning recultures and 5 were non-concerning. Among the concerning recultures, 1 was positive and 3 were negative. The graph highlights the scope reprocessing steps and the outcomes of the culturing process.

Navigate back to Figure 2.
