# Supplementary material for: Implementation and assessment of a postreprocessing endoscope surveillance program at the University of Kentucky, 2019–2024
Source: Infect Control Hosp Epidemiol. 2026 Apr 14;47(6):644–6. doi: 10.1017/ice.2026.10449 (PMC13216786; doi:10.1017/ice.2026.10449)
Supplement: Desmond et al. supplementary material [file S0899823X26104498sup001.docx]

**Supplemental material**

## **CRO surveillance and culture methods**

Endoscopic specimens were received in 90-mL aliquots of DEY-Engley (DE) broth, a neutralizing agent, containing both a swab and a brush. Upon receipt, each sample was vortexed for 20 seconds, after which the swab and brush were removed. The broth was then divided into two 50-mL conical tubes and centrifuged at 5,000 rpm for 15 minutes. The supernatant was discarded, and the resulting pellets were resuspended in a combined total volume of 0.4 mL of DE broth. A 0.1-mL aliquot of the suspension was plated onto Remel blood agar, MacConkey agar, MRSA chromogenic agar (Lenexa, KS), and Hardy ESBL chromogenic agar (Santa Maria, CA). Plates were examined at 24, 48, and 72 hours. Unused broth was tested for sterility, and an aliquot of the used DE broth was tested daily for inhibitory activity using Escherichia coli ATCC 25922.

Organisms were identified using the Bruker MALDI-TOF Clinical Application Database (Billerica, MA). Susceptibility testing for all high-risk gram-negative organisms was performed using the BD Phoenix Emerge panel (Sparks, MD). Isolates with elevated MIC values to carbapenem agents were confirmed for carbapenemase production using either the Hardy Diagnostics NG-CARBA-5 assay (Santa Maria, CA) or the modified carbapenem inactivation method (mCIM), as clinically indicated, in accordance with CLSI guidelines.

## **Low/moderate concerning vs High concerning organisms (1)**

High Concern Organisms –examples include but are not limited to staphylococcus auereus, enterococcus spp., streptococcus spp., including viridans group, pseudomonas aeruginosa, E. coli, Klebsiella spp., Salmonella spp., Shigella spp., and other enteric gram –negative bacilli as well as Candida spp.

Low or Moderate Concern Organisms –examples include but are not limited to coagulase negative staphylococci (not including staphylococcus lugdenensis), micrococci, diphteroids, Bacillus spp., ralstonia spp., Stenotrophomonas spp., and other water related organisms except for bukholderia and pseudomonas


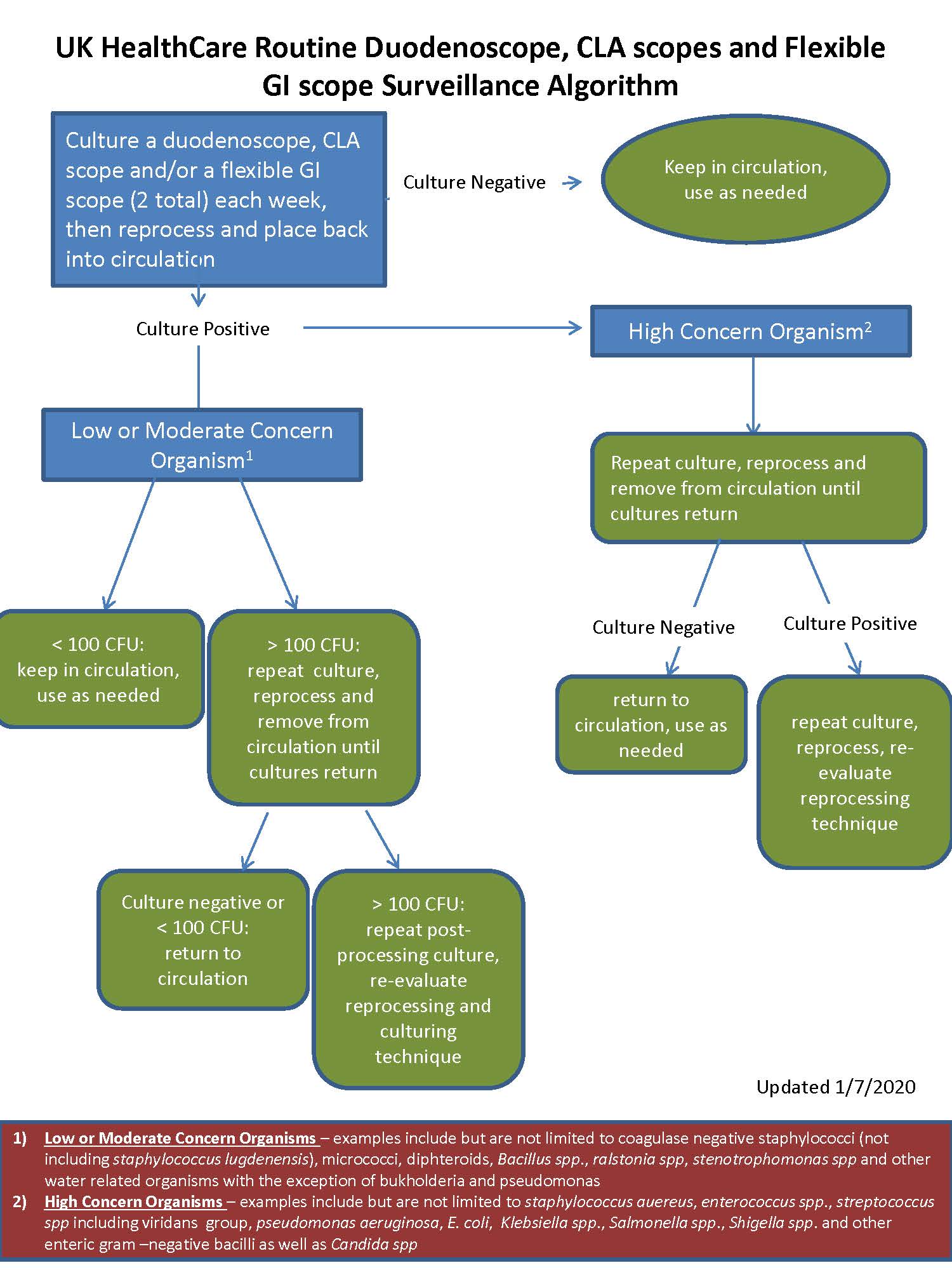


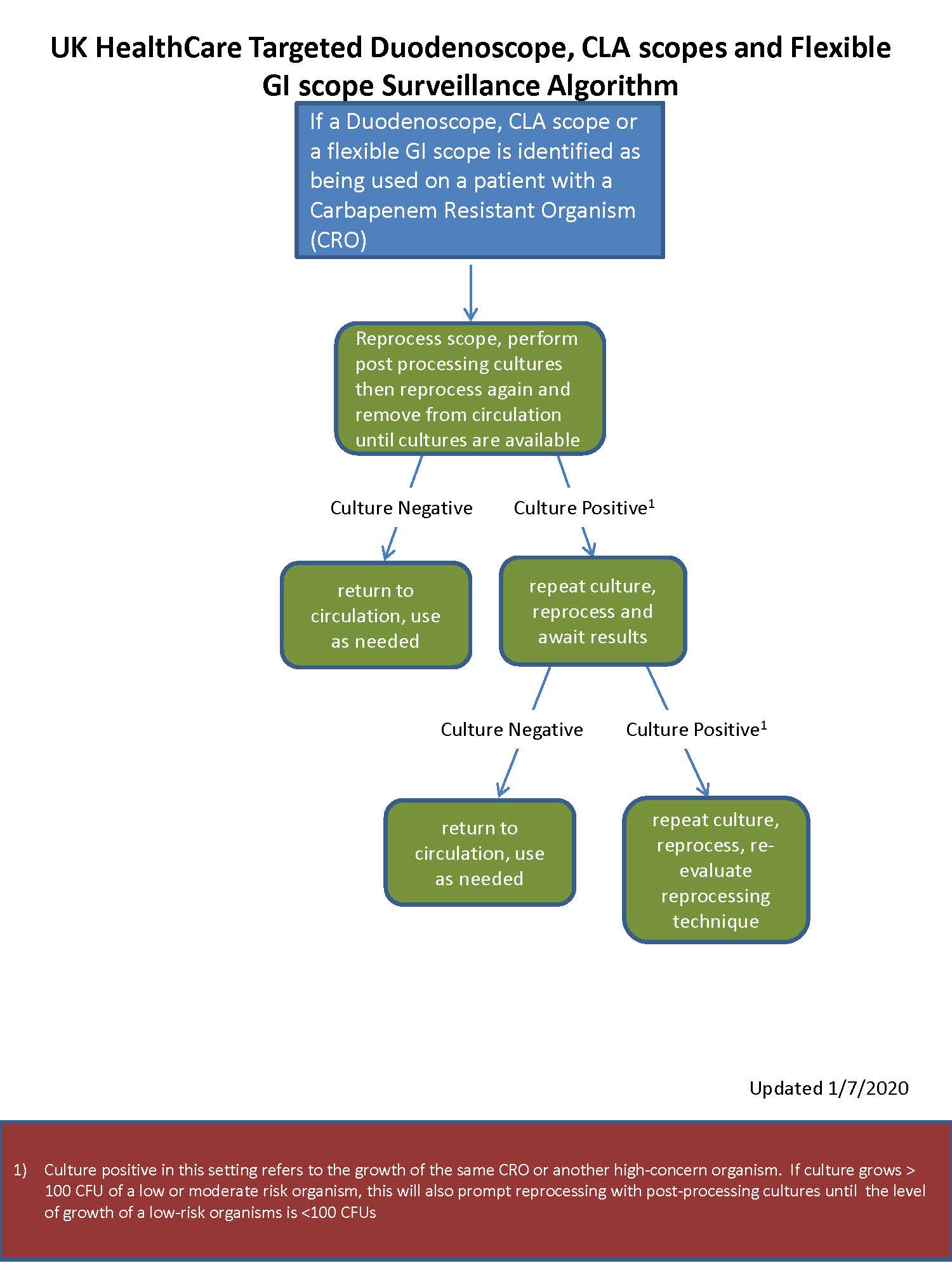


**Reference**

1. Prevention CfDCa. Interim Duodenoscope Culture Method. CDC: Atlanta, GA, USA. 2015.
